# The effect of chronobiology on frontal functions and social functionality in remitted patients with schizophrenia

**DOI:** 10.1192/j.eurpsy.2023.549

**Published:** 2023-07-19

**Authors:** B. Çınar, S. Gıca, N. Karamustafalıoğlu

**Affiliations:** ^1^psychiatry, Ardahan Public Hospital, Ardahan; ^2^psychiatry, Necmettin Erbakan Üniversitesi Meram Tıp Fakultesi, Konya; ^3^psychiatry, Istanbul Bakirkoy Prof. Dr. Mazhar Osman Research and Training Hospital for Psychiatry, Neurology and Neurosurgery, İstanbul, Türkiye

## Abstract

**Introduction:**

Due to the effects of sleep on the central nervous system,it is thought that sleep problems have a special importance in the onset, course and treatment of psychiatric diseases. Although the negative effects of sleep problems on the occurrence, recurrence and clinical course of psychiatric disorders are well known, it is reported that clinicians do not spend enough time for sleep problems in practice.To our knowledge there is no study in the literature which examining its effect on frontal lobe functions or social functionality in schizophrenia.

**Objectives:**

In current study, it is aimed i- to examine the chronobiological characteristics of remitted patients with schizophrenia, ii- to determine the effect of chronobiology on sleep quality, frontal lobe functions, depressive symptoms, interpersonal relationships and social functionality in patients with schizophrenia, iii- thus to improve the quality of life and the treatment outcome of patients with schizophrenia.

**Methods:**

185 patients with schizophrenia who met the Andreason remission criteria were included in the study. The patients were evaluated with Positive and Negative Syndrome Scale (PANSS), Morningness & Eveningness Scale (MEQ), Calgary Depression Index (CDI), Pitssburgh Sleep Quality Index (PSQI), Barratt Impulsivity Scale Short Form (BIS-11-SF), Frontal Assessment Battery (FAB), Personal Social Performance Scale (PSPS). Each patient was classified either as morning type (MT) or evening type (ET) or intermediate type (IT) according to MEQ scores.

**Results:**

The obtained MEQ responses indicated that 29 (15.7%) of the patients were “ET”, 124 (67.0%) were “IT”, and 32 (17.3%) were “MT”. In the “ET” group, the mean of CDI total score, the mean of PSQI total score, the mean of BIS-11-SF total and the mean score of BIS-11-SF planlessness and inattention subscale were higher than in both the “IT” and “MT”. The mean total scores PSPS of “ET” patients were found to be lower than the mean scores of both the “IT” and “MT” patients. There was no significant difference between the groups in terms of FAB total scores. According to Multiple Linear Regression Analysis, total MEQ score and PANSS-negative symptoms subscale score were found to have an effect on PSPS[1]Personal Social Realitionship subscale scores.

**Conclusions:**

Although all patients included in current study were in remission, it was found that chronotype characteristics were effective on many clinical manifestations and comorbid conditions. The findings obtained from our study emphasize how important it is to question chronotypic features in daily psychiatric practice. In this context, being aware of the importance of chronotypic features in the treatment of schizophrenia patients, questioning the patients in this respect and taking necessary interventions may have the potential to improve functionality, which is one of the main treatment goals in patients with schizophrenia.

**Disclosure of Interest:**

None Declared

